# Genome-Wide Identification and Expression Analysis of Auxin Response Factor (ARF) Gene Family in Longan (*Dimocarpus longan* L.)

**DOI:** 10.3390/plants9020221

**Published:** 2020-02-08

**Authors:** Yuan Peng, Ting Fang, Yiyong Zhang, Mengyuan Zhang, Lihui Zeng

**Affiliations:** 1 College of Horticulture, Fujian Agriculture and Forestry University, Fuzhou 350002, China; pengyuan456@outlook.com (Y.P.); fangting@fafu.edu.cn (T.F.); zhangyiyong66@163.com (Y.Z.); zy15659121197@126.com (M.Z.); 2 Institute of Genetics and Breeding in Horticultural Plants, Fujian Agriculture and Forestry University, Fuzhou 350002, China

**Keywords:** longan, ARF, bioinformatics analysis, quantitative analysis, flowering

## Abstract

Auxin response factor (ARF) is the key regulator involved in plant development. Despite their physiological importance identified in various woody plants, the functions of ARF genes in longan were still not clear. In this study, 17 longan ARF genes (*DlARF*) were identified using the reference longan genome data. According to the phylogenetic relationships among longan, *Arabidopsis* and apple, DlARFs were divided into four classes. Most DlARFs showed a closer relationship with ARFs from apple than those from *Arabidopsis*. The analysis of gene structure and domain revealed high similarity of different ARF genes in the same class. Typical features of B3-type DNA binding domain (DBD) motif, Auxin Resp motifs, and a highly conserved C-terminal Phox and Bem1 (PB1) domain were present in all DlARFs except for *DlARF-2,-3,-13* which lacked PBI domain. Expression profiles of 17 DlARF genes in longan different tissues showed that some DlARF genes were tissues-specific genes. Analysis of three longan transcriptomes showed seven *DlARFs* (*DlARF-1,-2,-6,-8,-9,-11,-16*) had higher expression levels during floral bud differentiation of common longan and in the buds of ‘Sijimi’, suggesting these genes may promote floral bud differentiation in longan. Further qPCR analysis showed that among seven DlARF genes, the expression levels of *DlARF-2,-6,-11,-16* increased significantly during the physiological differentiation stage of longan floral buds, confirming that they may play a role in flowering induction. Promoter sequence analysis revealed cis-elements related to flowering induction such as low-temperature responsiveness motif and circadian control motif. Motifs linked with hormone response for instance Auxin, MeJA, Gibberellin, and Abscisic acid were also found in promoters. This study provides a comprehensive overview of the ARF gene family in longan. Our findings could provide new insights into the complexity of the regulation of ARFs at the transcription level that may be useful to develop breeding strategies to improve development or promote flowering in longan.

## 1. Introduction

Auxin is a vital regulator of plant growth, and plays important roles not only in diverse aspects of plant development such as control of leaf vascular patterning [1], apical dominance [2], establishment of embryonic axis [3], and root geotropism phototropism of the stem [4], but also in fruit development and controlling circadian flower opening and closure [5,6]. Indeed, different growth regulators influenced flower formation, which are dependent on the physiological stage of the explant, particularly its endogenous hormone levels [7]. Previously, the floral transition will be initiation by an endogenous increase in the levels of indole-3-acetic acid (IAA) influenced by Thidiazuron of *Dendrobium* [8]. Over the past 30 years, it has been clearly certificated that auxins can exert rapid and specific effects on genes at the molecular levels [9]. Many gene families, such as auxin/indole-3-acetic acid (Aux/IAA), gretchen hagen 3 (GH3), small auxin-up RNA (SAUR), and auxin response factor (ARF), were involved in auxin signal transduction, forming a complex auxin signal regulation network at the molecular level [10]. In this regulatory pathway, ARF is an important transcription factor family, which DNA binding domain (DBD) can specifically bind to TGTCTC auxin response elements (AuxREs) in the target gene promoter to participate in auxin signal transduction and regulate auxin responsive genes expression [11]. A working model for ARF activation responses to auxin is now well-established [12]. Under lower auxin concentration, Aux/IAA proteins form dimers with ARFs proteins inhibit ARF activity by recruiting the co-repressor TOPLESS (TPL), which results in the repression of auxin responsive genes [13]. At higher auxin concentration, Aux/IAAs proteins bind to the SCFTIR1/AFB complex and rapidly become ubiquitinated and degraded, allowing ARFs to release and activate transcription of its target genes [14].

Considering the roles of ARFs in various physiological and developmental, ARF gene families have been identified and characterized in many plants such as *Arabidopsis thaliana*, potato (*Solanum tuberosum*), rice(*Oryza sativa*), papaya (*Carica papaya*), and chickpea (*Cicer arietinum*) [15,16,17,18,19]. Although biochemical and genetic analyses have established a crucial function for *ARF* genes in plant growth and development, current knowledge of the biological roles of individual ARFs is mainly from the characterization of model plants Arabidopsis, rice and tomato. In *Arabidopsis*, *ARF2* regulates leaf senescence and floral organ abscission independently of the ethylene and cytokinin response pathways [20]; *ARF6*, *NPH4/ARF7*, and *ARF8* affect leaf and inflorescence growth, promoting hypocotyl elongation [21,22]. In rice, negative regulation of *OsARF18* expression by *OsmiR160* is critical for rice normal growth and development [23]; *OsARF12* is regarded as one of major player in phosphate-induced auxin responses, indicating that ARFs might be involved in phosphate homeostasis in crops [24]. In tomato, *SlARF7* acts as a negative regulator of fruit set after pollination and fertilization, and moderates auxin response during fruit growth [25]; *SlARF4* controls chlorophyll accumulation in fruit and down-regulation of *SlARF4* results in a dark-green immature fruit phenotype with increased chloroplasts [26]. In addition, genome-wide identification and expression analyses of the ARF family suggest that the expression of many *ARF* genes is altered in various species in responsive to abiotic stresses, such drought, salt, or cold [27,28]. Together, ARF family genes play an important role in regulating plant growth and development, and in plant responses to multiple signaling transduction pathways. However, to our knowledge, ARF gene family of longan (*Dimocarpus longan*) still remains unexplored.

Longan is an important subtropical fruit tree of Sapindaceae family, which is grown in many subtropical and tropical countries with most of the production in Southeast Asia and Australia [29]. As an edible drupe fruit and source of traditional medicine, longan is grown in most areas of Southern China, including Guangdong, Guangxi, Fujian, Sichuan, Yunnan, and Hainan [30]. Considering the significance of ARFs in plant growth and development, information on ARF gene family of longan is needed for better understanding. In this study, a comprehensive analysis of ARF family genes was conducted in longan based on its genomic sequence and three transcriptome databases [31,32,33]. A total 17 *Dimocarpus longan* ARF (DlARF) genes were identified and their physical and chemical properties, conserved motifs, genomic structures, evolutionary relationship and functional classification were analyzed. Moreover, expression and promoter cis-acting elements of DlARF genes were investigated. Our results will provide valuable information for understanding the classification and putative functions of DlARFs and make more insight into the molecular basis of auxin responses in longan.

## 2. Materials and Methods

### 2.1. Plant Materials

Longan (cultivar ‘Honghezi’) plants are in experimental field of Fujian Agriculture and Forestry University. The shoot tips from branches that were expected to bloom were collected monthly over a period of 4 months from October 2018 to January 2019. The samples were stored at −80 °C.

‘Honghezi’ seedlings were grown for six month, which were used for hormonal treatments. Plants were treated with abscisic acid (ABA, 50 mM), methyl jasmonate (MeJA, 100 mM), gibberellic acid (GA_3_, 50 mM), and 6-benzylaminopurine (6-BA, 75 mM) for 4 h at 26 ± 2 °C, respectively. Five plants were used in each treatment. Meanwhile, five seedlings have no treatments as a control. The leaves were immediately frozen in liquid nitrogen and stored at −80 °C until RNA extraction. Collect three samples per treatment and each sample was made by pooling at least five plants.

### 2.2. Identification of ARF Genes in Dimocarpus Longan

The *Arabidopsis* ARFs (AtARFs) gene sequences were downloaded from NCBI gene database (https://www.ncbi.nlm.nih.gov) according to the Gene ID [9], the longan genomic were obtained from GigaScience (http://gigadb.org) and the apple (*Malus domestica*) ARFs family were loaded from GDR Gene Database (https://www.rosaceae.org). The *Arabidopsis* ARFs protein sequence were used as queries to search against longan genome and the putative genes were identified based on a local BLASTP search at the score value of e-30.0 value. The 17 longan ARF genes were further confirmed by the hidden Markov model (HMM) profiles of the ARF gene family which include B3 DNA binding (Pfam 02362), Auxin Resp (Pfam 06507), and PB1 domains (Pfam 02309). The molecular weight (MW) and isoelectric points (PI) of longan ARF family proteins were obtained from ExPASy tool [34], and the Amino-acid content of the MR domain in DlARFs was calculated using InterPro (http://www.ebi.ac.uk/interpro).

### 2.3. Sequence Alignment and Phylogenetic Tree

Multiple alignment of ARF proteins were analyzed by MUSCLE progress of MEGA6.0. Based on this alignment, a bootstrapped neighbor-joining (NJ) tree was constructed using MEGA (version 6.0) with the bootstrap test replicated 1000 times [35]. Classified and annotated of ARF protein sequences by using iTOL (https://itol.embl.de/itol.cgi).

### 2.4. Gene Structure and Domain Analysis of DlARFs

Conserved domains of DlARF proteins were identified using the InterPro, the original annotation file in gff3 format of longan genome was downloaded from GigaScience (http://gigadb.org), CDS and UTR domain of DlARFs were analyzed and annotated by TBtools [36]. The cis-acting elements of DlARFs gene promoter were analyzed using PlantCARE [37].

### 2.5. RNA Isolation and Quantitative Real Time (qRT-PCR)

RNA was extracted with EASY spin plus complex plant RNA kit provided by Aidlab company (Beijing, China), reverse transcription RNA into a single-stranded cDNA by using the PrimeScript^TM^ RT reagent kit and gDNA Eraser kit provided by TaKaRa. The primers were designed by DNAMAN8.0 software and tested to ensure amplification of single discrete bands with no primer-dimers. Product size from 140bp to 200bp. Primer sequences are shown in detail in the Supplementary Table S1. Longan *actin* gene was used as the reference gene for expression analyzing [38]. The qRT-PCR experiment used CFX96 real-time PCR Detection system (Bio-Rad laboratories, Hercules, CA, USA) and TB Green mixture (TaKaRa Bio, Shiga, Japan). The relative expression of specific gene was quantitated with the 2^−ΔΔCt^ calculation method [39].

## 3. Results

### 3.1. Identification of DlARF Gene Family in Longan

**Figure 1:**
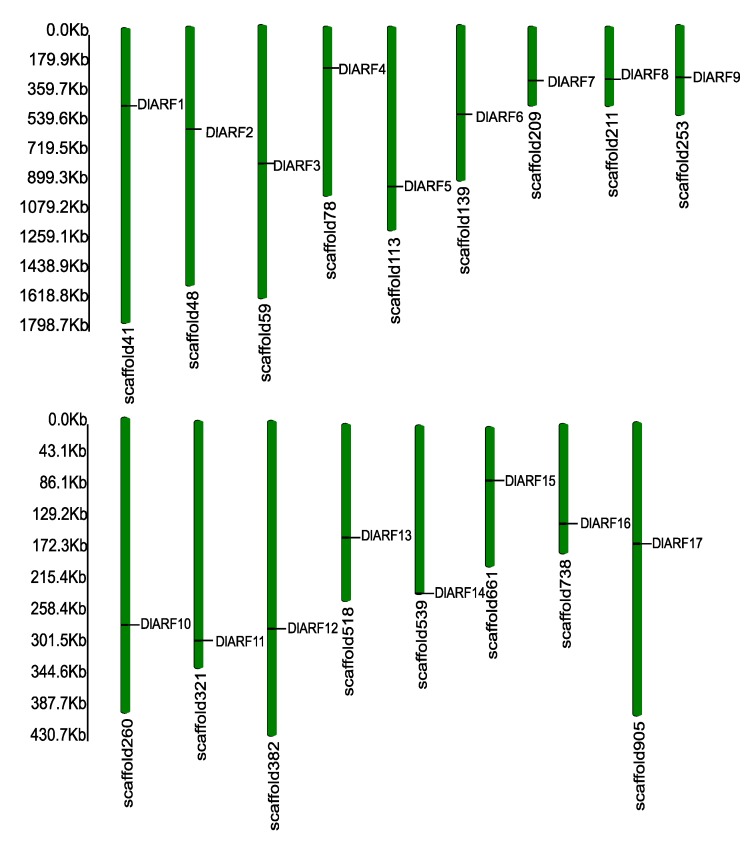
The location in scaffold of *Dimocarpus longan* auxin response factor (DlARF) family. The scaffold numbers and size are indicated at the top and bottom of each bar, respectively. The scale is in kilo bases (kb).

A BLASTp search was implemented against the longan genome using Arabidopsis ARF genes as queries to perceive putative genes encoding the ARF. A total 17 ARF genes were identified in longan. All the high-quality reads of longan were assembled into 51,392 contigs and 17,367 scaffolds (≥200 bp) [31], 17 ARF genes were concentrated in the scaffold range from number 41 to 905. According to their position from the top to the bottom on the scaffold, 17 ARFs were named from DlARF1 to DlARF17 (Figure 1).

Physical and chemical properties of 17 DlARF genes were listed in Table 1, including isoelectric point (PI), molecular weight (MW) and coding sequence length (CDS). The length of 17 DlARF proteins varied from 575 (DlARF3) to 1498 aa (DlARF6), with an average of 845 aa. They had the lowest MW of 63.54 KDa (DlARF3) and the highest MW of 132.00 KDa (DlARF1), with an average of 93.86 KDa. The PI varied from 5.27 (DlARF16) to 8.03 (DlARF14), with an average of 6.39. Furthermore, all DlARF proteins are unstable, hydrophobic protein, suggesting that they might play roles in different subcellular environments.

### 3.2. Phylogenetic Analysis and Classification of DlARF Genes

**Figure 2:**
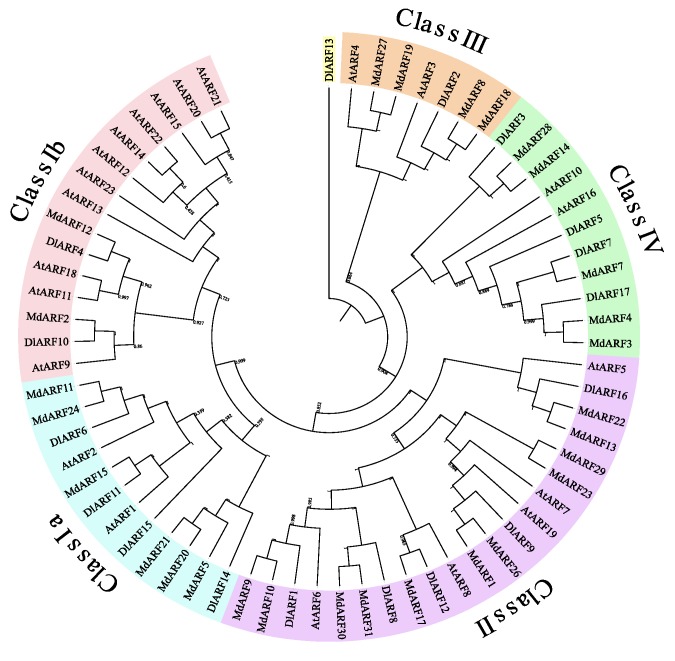
The phylogenetic analysis of ARF family genes of *Arabidopsis*, apple, and longan. Tree was constructed via neighbor-joining method with 1000 bootstrap replications.

To investigate the phylogenetic relationship of DlARFs, the neighbor-joining (NJ) method [35] was used to construct a phylogenetic tree consisting of 17 DlARFs, 21 AtARFs of *Arabidopsis* and 31 MdARFs of apple. The phylogenetic distribution indicated there were higher homologous between longan and apple, possibly due to they are both woody fruit trees, however, there still more than 50% homology between longan with *Arabidopsis*. All ARFs analyzed were divided into four classes (classⅠ, classⅡ, classⅢ, and classⅣ) (Figure 2). ClassⅠ contained the most ARF numbers (6 DlARFs, 8 MdARFs and 13 AtARFs) and was further divided into two subgroups. ClassⅡ included five DlARFs. ClassⅢ and classⅣ included one and four DlARFs, separately. DlARF13 was in an isolated branch which lacks of *Arabidopsis* orthologs.

### 3.3. Structure Analysis and Motif Composition of DlARF Genes

**Figure 3:**
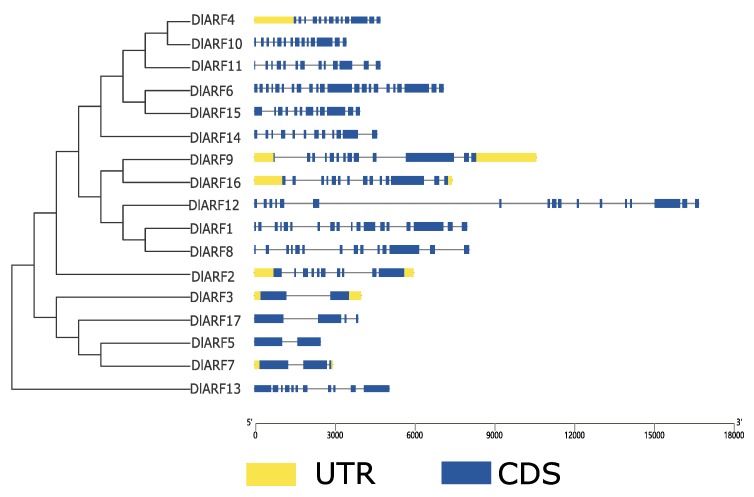
Gene structure analysis of DlARFs family genes. The blue boxes, yellow boxes, and the black lines indicate upstream/downstream, exons, and introns, respectively.

By comparing the corresponding genomic DNA sequences, exon and intron structures of DlARF genes were obtained. The results showed that different classes contained different exon numbers, ranging from 2 to 22 (Figure 3). For instance, there were 12–22 exons in *DlARF-4,-6,-10,-11,-14,* and *-15* which were all grouped in classI. There were 13–18 exons in *DlARF-1,-8,-9,-12* and *DlARF-16* which belonged to classⅡ. *DlARF2* in classⅢ contained 18 exons, and *DlARF-3,-5,-7,-17* in class Ⅳ had the smallest number of exons which ranged from 2 to 4.

**Figure 4:**
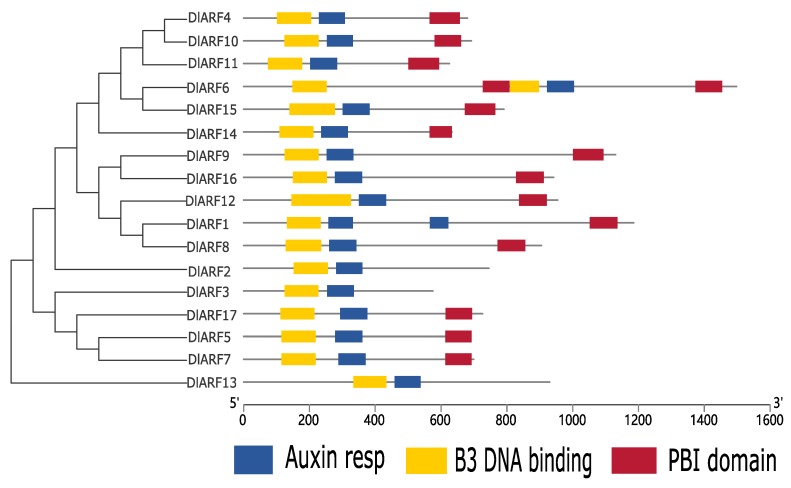
Analysis of conserved domain in DlARFs proteins. All conserved domains were identified by MEME database with the complete amino acid sequences of DlARFs.

In addition to structural analysis, the pattern of conserved domains between ARF subclasses can also provide some clues on classification (Figure 4). It was reported that the typical features of ARF protein are B3-type DBD motif, Auxin Resp motifs, and a highly conserved C-terminal PB1 domain [15]. Our results showed that most of DlARF proteins had these three typical domains except for DlARF2 in class Ⅲ, DlARF3 in class Ⅳ, and DlARF13 which lacked C-terminal PB1 domain.

### 3.4. Expression Profiles of DlARF Genes in Different Plant Tissues

**Figure 5:**
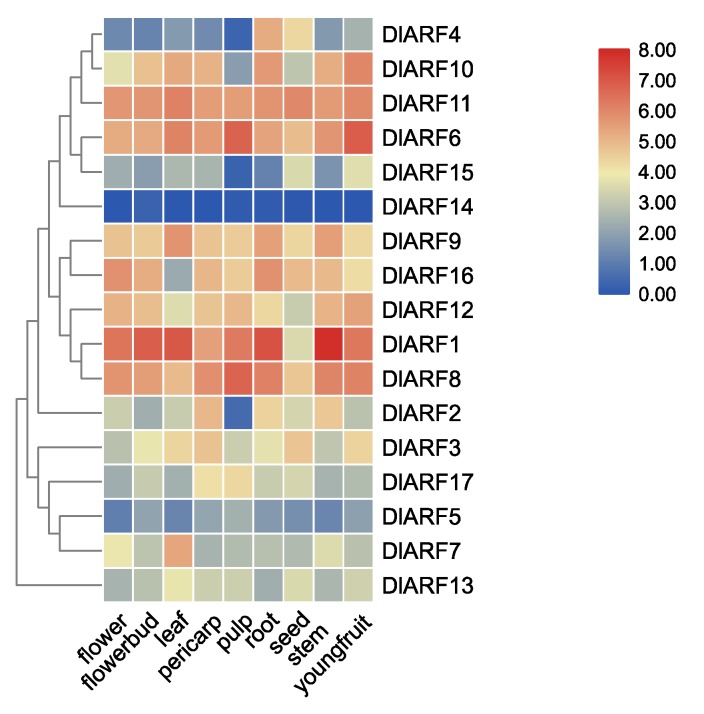
Expression analysis of DlARF genes in different tissues of longan.

To explore the putative functions of *DlARFs*, FPKM (Fragments per Kilobase of transcript per million mapped reads) values were used to assess the expression pattern of 17 *DlARF* genes in nine different tissues of longan (Figure 5). The expression levels of *DlARF-1,-6,-8,-11* were ubiquitously abundant in all tissues. *DlARF1* had higher expression in stems and roots, *DlARF6* had the higher expression levels in young fruits and pulp, *DlARF8* had the highest expression level in pulp, whereas *DlARF5* and *DlARF14* were absent from nine tissues (FPKM value < 3). *DlARF-3,-17,-13,-15* had lower expression levels in nine tissues (FPKM value < 30). *DlARF-2,-4,-7* were tissue-specific genes, *DlARF2* showed pericarp-specific expression, *DlARF4* showed root-specific expression, while *DlARF7* was leaf-specific gene.

### 3.5. Expression Profiles of DlARF Genes in Different Longan Varieties

**Figure 6:**
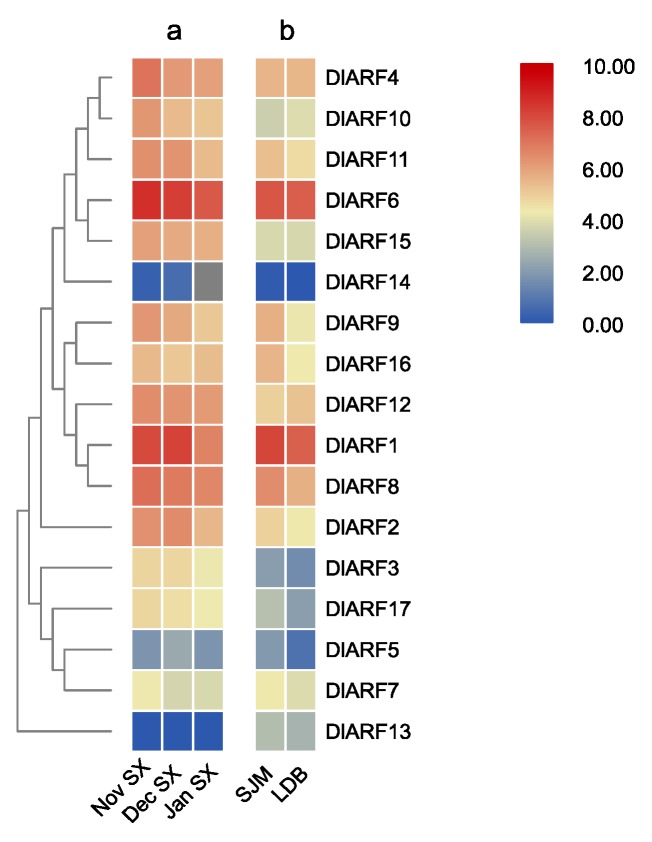
Expression analysis of *DlARF* genes from different varieties. (**a**): expression of *DlARFs* in apical buds of ‘Shixia’ from November to January of the following year; (**b**): expression of *DlARFs* in the buds of the ‘Lidongben’ and ‘Sijimi’. Note: SX is Shixia, SJM is Sijimi, LDB is lidongben.

‘Shixia’ and ‘Lidongben’ are common longan varieties exhibiting seasonal flowering traits, while ‘Sijimi’ is a specific variety with continuous flowering trait. Firstly, the expression patterns of 17 *DlARF* genes were analyzed using RNA-seq databases of apical buds during floral bud development in ‘Shixia’ [33]. In Guangdong Province of China, apical buds in November are at the stage of before the emergence of floral primordia, apical buds in December are at the stage of floral organ differentiation which is characterized by the appearance of red dot, and apical buds in January is characterized by the appearance of the first inflorescence [33]. The results showed that there were nine *DlARFs* (*DlARF-1,-2,-4,-6,-8,-9,-10,-11,-16*) that showed higher transcript levels in November and December in ‘Shixia’ (Figure 6a). In addition, the expression levels of 17 *DlARFs* were compared between ‘Sijimi’ and ‘Lidongben’ using the RNA-seq databases, which were collected from apical buds of grafting newly-sprouting [32] (Figure 6b). The results showed that seven *DlARFs* (*DlARF-1,-2,-6,-8,-9,-11,-16*) had higher expression levels in ‘Sijimi’ than in ‘Lidongben’. Taken together, seven genes (*DlARF-1,-2,-6,-8,-9,-11,-16*) showed higher expression in both analyses.

### 3.6. Expression Patterns of Seven DlARF Genes during Floral Bud Physiological Differentiation of Longan

**Figure 7:**
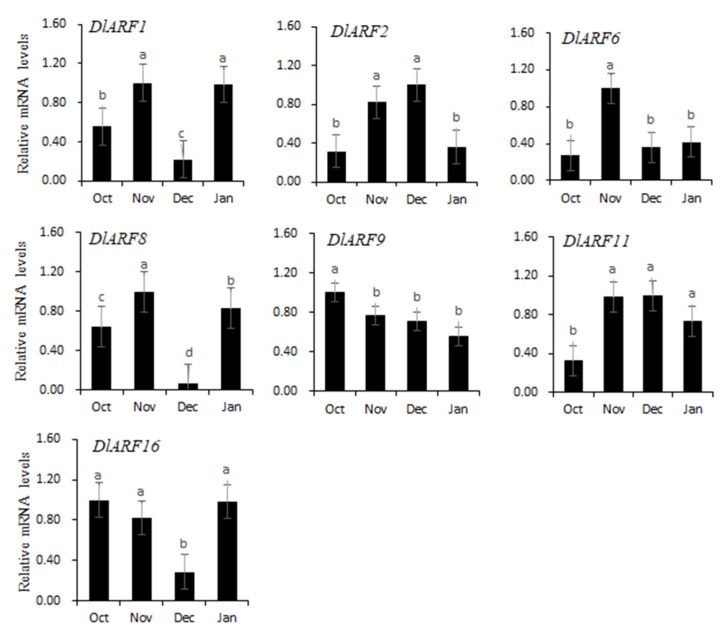
Expression patterns of seven *DlARF* genes during floral bud physiological differentiation of longan. The x-axis indicates months and the y-axis indicates the relative gene expression. Values with the different letter (a, b, c) were significantly different when assessed using Duncan’s multiple range test (*p* < 0.05).

Real-time qPCR was performed further to explore the expression patterns of seven *DlARF* genes (*DlARF-1,-2,-6,-8,-9,-11, -16*) during floral bud physiological differentiation of longan (Figure 7). In Fujian Province, China, the physiological differentiation stage of longan floral buds is in November and December [40]. The expression of *DlARF-2,-6,-11* showed a trend of increasing first and then decreasing, peaking in the November or December. *DlARF16* showed the opposite tendency, the expression level decreased in December and increased significantly in January. The transcript levels of *DlARF1* and *ARF18* did not change obviously from October to January.

### 3.7. Cis-Element Prediction in Promoters of Seven DlARF Genes

The distribution of cis-elements may reflect the potential up-stream regulatory factors of genes. Thus, the 1500 bp upstream sequence of *DlARF-1,-2, -6, -8, -9,-11, -16* was obtained from the longan genome and analyzed, the putative cis-elements of these promoters were shown in Table S2.

Cis-elements related to circadian control were found in *DlARF-6,-8,-9,-11* promoters. In addition, an LTR motif related to low-temperature responsiveness was also found in the promotor of *DlARF-1,-8,-9*. Among seven promoters, five promoters *(DlARF-6,-8,-9,-11,-16)* contained one or more AuxREs motif. CGTCA/TGACG motif involved in MeJA responsiveness was found in the promotor sequence of *DlARF-6,-8,-11,-13,-16*. *DlARF-1,-6* had GARE-motif, which involved in gibberellin responsive. The promoter of *DlARF-1,-2,-6,-9,-16* had ABRE motif, which involved in abscisic acid responsiveness.

### 3.8. Expression Patterns of Seven DlARFs with Hormone Treatments

**Figure 8:**
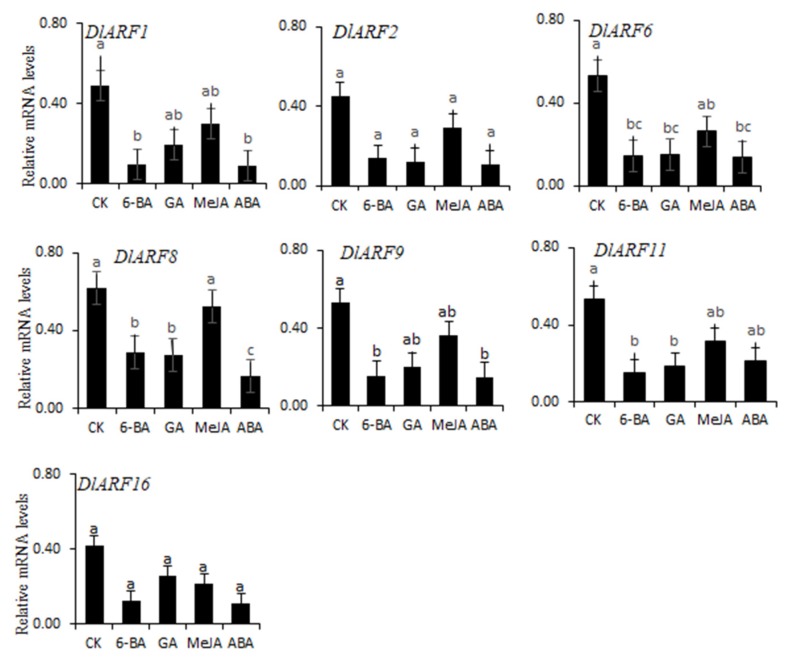
Expression patterns of *DlARF* genes under various hormonal stresses. The *x*-axis indicates various treatments and the y-axis indicates the relative gene expression; (*p* < 0.05, *n* = 3). Values with the different letter (a, b, c) were significantly different when assessed using Duncan’s multiple range test (*p* < 0.05).

Hormones play a very important role in the process of flowering [41]. Hormone-response motifs were found in the promoters of *DlARFs*, therefore the expression of seven *DlARF* genes in response to hormone treatments was investigated by qRT-PCR (Figure 8). The results showed that the expression levels of all seven *DlARF* genes declined in response to different hormone treatments. *DlARF-1,-6,-9,-11* had a significant decrease in expression levels.

## 4. Discussion

Auxin influences developmental processes from embryogenesis to senescence in plants, including that causing apical dominance, controlling embryo development, and promoting the floral transition [41,42,43]. ARFs is one of the important transcriptional regulator families to regulating the auxin transduction [15,44].

In this study, 17 *DlARFs* were identified in longan. The comprehensive phylogenetic analysis showed that longan ARFs could be divided into four subgroups, the same as previous phylogenetic classifications of ARFs in other species such as apple, banana [18], *Arabidopsis* [15], and rice [17].

The identification and classification of the DlARF gene family was supported by gene structure and conserved domain analyses. ARF proteins in higher plants are composed of three modular and portable domains, which range in size from 69 to 129 kDa and are defined by their N-terminal DBD [45]. The typical ARF protein contains a conserved N-terminal B3-type DBD, which is required for efficient binding to AuxRE [9], a variable middle region (MR) that functions as an activation domain (AD) or repression domain (RD), and a carboxyl-terminal Phox and Bem1 (PB1) domain (CTD: domain III/IV), which is involved in protein–protein interactions by dimerizing with auxin/indole-3-acetic acid (Aux/IAA) family genes as well as ARFs [15,46]. All DlARFs have a typical DBD domain. Additionally, most DlARFs contain a CTD domain, suggesting they can form dimers with ARFs or Aux/IAA proteins.

ARF binds to AuxRE motif in the promoters of auxin-regulated genes by activating (AD domain) or repressing (RD domain) which depend on a middle domain (MR) [47]. In *Arabidopsis*, the AD is enriched in glutamine (Q), leucine (L) and serine (S) residues and the RD is enriched in glycine (G), leucine (L), serine (S), and proline (P) residues [48]. The detailed protein sequence analysis revealed that the glutamine (Q), leucine (L) and serine (S) regions were found in the MR of *DlARF-1,-7,-9,-12,-16*, indicating these genes were more likely acting as activators. On the other hand, *DlARF-2,-6,10,-11,-13,-15* and DlARF17 were found to be rich in the glycine (G), leucine (L), serine (S), and proline (P) regions rich in the MR, indicating they are more likely acting as repressors. The activator/repressor ratio among DlARFs is 0.35, which is higher than that in Medicago (0.26) but lower than that in Arabidopsis (0.59). However, the mechanisms of activation and repression are still unclear [49].

Tissue expression analyses revealed that 17 *DlARF* genes had different expression patterns in different tissues. *DlARF-6,-10,-11* which were clustered into classⅠ showed higher expression in leaf, pulp, and young fruit. In *Arabidopsis*, the functions of ARF genes in the same class were involved in controlling leaf senescence and floral organ abscission [20], so *DlARF-6,-10,-11* might have similar functions in regulating leaf senescence. *DlARF-1,-8,-9,-12,-16* in classⅡ showed higher expression in stem, root, and flower, there have been reported that *AtARF* genes in classⅡ played an important role in plant growth and development, such as regulating flower maturation and fertilization, promoting fruit development [22,50], therefore, they might also play an important role in flower growth. *DlARF2* in classⅢ and *DlARF-3,-5,-7,-17* in classⅣ showed lower expression in all nine tissues. There were few studies in the same category genes in *Arabidopsis* or apple, their functions remain unclear. *DlARF13*, a special gene, was clustered in an isolated class. DlARF13 shares 35% identity at the amino acid level with the *Citrus sinensis* ARF14 (*Cs7g02210.1*), which was also clustered to an isolated class [27] (Figure S1). This separated class is absent from herbaceous annual plants, such as *Arabidopsis*, cassava, and rice, but present in sweet orange and other woody perennials, so it might be a woody preferential specific clade [15,17,19].

It has been reported that free endogenous IAA was intimate correlated with the early stage of flower bud differentiation, high concentration of IAA, which act in apical bud will promote flowering. [36,51,52,53]. Seven *DlARFs* genes (*DlARF-1,-2,-6,-8,-9,-11,-16*) have been identified to show higher expression levels during longan floral bud induction and flower organ development. At the same time, these seven genes showed higher expression in the grafting newly-sprouting buds of ‘Sijimi’. ‘Sijimi’ has a continuous blossoming trait, the newly-sprouting buds in ‘Sijimi’ are speculated to have the capacity of continuous flowering after maturity [32]. These results suggest that these seven *DlARF* genes may play a role in promoting floral bud differentiation in longan. According to previous research, down-regulation of *SlARF6* and *SlARF8* by *miR167* leads to floral development defects and female sterility in tomatoes. *SlARF6* and *SlARF8* have conserved roles in controlling growth and development of vegetative and flower organs in dicots [54]. *DlARF8* and *DlARF1*, which were similar with tomato *SlARF6* and showed a high expression in flowers, may have the same roles in controlling flower organ development.

Further analysis showed that the expression levels of *DlARF-2,-6,-11,-16* increase significantly during the physiological differentiation stage of floral buds, indicating they might play an important role in flowering induction. In *Arabidopsis*, *AtARF2* mRNA are present in roots, leaves and flowers [48], and promotes transitions between multiple stages of development [20,22]. *DlARF6*, a homologous gene of *AtARF2*, which expression increased during flower bud physiological differentiation period, peaked in November, may be similar with *AtARF2*, participating in the regulation of flowering transition. In *Arabidopsis*, *AtARF1* acts in a partially redundant manner with *AtARF2* [20]. The transcript of *DlARF11* (homolog gene of *AtARF1*) also was abundant in the buds during the physiological differentiation period, suggesting it may have partially redundant manner with *DlARF6*. *AtARF3* not only integrated the function of *AGAMOUS (AG)* and *APETALA2 (AP2)* in floral meristem determinacy [55], but also regulated floral meristem determinacy by repressing cytokinin biosynthesis and signaling [56]. *DlARF2* (similar to *AtARF3*), showed high expression level during the flower bud differentiation, and it also highly expressed during the physiological differentiation stage, suggesting that *DlARF2* might have the same function in floral meristem determinacy. *AtARF5* (the homologous gene of *DlARF16*), is critically required for embryonic root and flower formation [57]. *DlARF16* showed a higher expression level in flower and root, so it might play a role in root and flower development of longan.

Environmental conditions and endogenous circadian rhythms adjust the flowering time and thereby enable plants to reproduce. Low temperature is required for flowering induction in longan. Cis-elements analysis reveals some flowering-related motifs such as low-temperature respond and circadian control [58], suggesting these *DlARFs* response to low temperature and circadian rhythms and then regulate flowering. There was also an enrichment of cis-elements linked with hormone response. Many hormones have been reported to be involved in the regulation of flower opening or formation with relation to both internal and external cues, such as gibberellic acid, which is able to induce flowering at low temperature and control onset of flower formation in *Arabidopsis*. Jasmonic acid facilitates flower opening and floral organ development through the up-regulated expression of *SlMYB21* in tomato [59,60,61]. The result of real time PCR experiment revealed seven *DlARFs* responded to cytokinin, MeJA, Gibberellin, and Abscisic acid. In potato, the expression of *StARFs* under various phytohormone treatments (GA3, ABA, auxin, cytokinin) were analyzed, most *StARF* members slightly decrease or even unchanged in response to hormones [16]. In sweet orange, *CiARFs* present at low levels at 6 h with the IAA treatment and up-regulated at 12 h by IAA and NPA treatments [27]. Our results are similar with *CiARFs* in response to 6 h IAA treatment.

## Figures and Tables

**Table 1:** Physical and chemical properties of ARFs in longan.

Gene Name	Gene Identifier	Exon Numbers	PI	MW (kDa)	Genomic Position	ORF Length (bp)	Homolog in Arabidopsis	MR Domain (Amino Acid Content)
DlARF1	Dlo.020240.1	18	6.66	132.00	scaffold41:473073-481372	3558	AtARF6	QSL
DlARF2	Dlo.022331.1	18	6.61	80.87	scaffold48:620922-626016	2238	AtARF3	GLS
DlARF3	Dlo.025765.1	2	5.76	63.54	scaffold59:841815-845259	1728	AtARF10	PSV
DlARF4	Dlo.030332.1	13	6.83	76.01	scaffold78:251174-254549	2043	AtARF11.18	LSV
DlARF5	Dlo.002610.1	2	6.48	72.72	scaffold113:973236-975819	1986	AtARF16	GLS
DlARF6	Dl0.005415.2	22	6.15	166.68	scaffold139:542989-550375	4497	AtARF2	EPS
DlARF7	Dlo.011077.1	2	6.58	77.10	scaffold209:327655-330450	2100	AtARF16	QSL
DlARF8	Dlo.011327.1	14	5.89	100.03	scaffold211:316240-324625	2718	AtARF6	QSL
DlARF9	Dlo.013714.2	13	6.10	126.04	scaffold253:313870-321781	3393	AtARF19	QSL
DlARF10	Dlo.014083.1	14	6.05	77.38	scaffold260:282418-286013	2079	AtARF9	LSV
DlARF11	Dlo.016820.1	12	5.96	69.86	scaffold321:299097-304013	1878	AtARF1	PSV
DlARF12	Dlo.019200.4	17	6.22	106.61	scaffold382:283090-300432	2850	AtARF8	QSL
DlARF13	Dlo.023607.1	11	5.99	104.31	scaffold518:150916-156184	2793		LSV
DlARF14	Dlo.024143.1	12	8.03	70.51	scaffold539:226240:231034	1902		GLS
DlARF15	Dlo.027785.3	12	6.87	88.20	scaffold661:72501:76620	2376	AtARF2	LSV
DlARF16	Dlo.029636.1	14	5.27	104.34	scaffold738:134978:141446	2829	AtARF5	QSL
DlARF17	Dlo.032913.1	4	7.21	79.38	scaffold905:165555-169610	2181	AtARF16	GLS

Note: Q, glutamine; S, serine; L, leucine; P, proline; G, glycine; E, glutamic acid; V, Valine.

## Supplementary Materials

The following are available online at https://www.mdpi.com/2223-7747/9/2/221/s1, Table S1: The primer sequences of seven DlARFs and reference gene in longan, Table S2: Analysis of cis-elements in flower bud differentiation related ARF genes in longan, Figure S1: Protein sequences alignment between CiARF14 and DlARF13.
