# Supplementary material for: Genome-Wide Identification and Expression Analysis of Auxin Response Factor (ARF) Gene Family in Longan (Dimocarpus longan L.)
Source: Plants (Basel). 2020 Feb 8;9(2):221. doi: 10.3390/plants9020221 (PMC7076634; doi:10.3390/plants9020221)
Supplement: Supplementary file 1 [file plants-09-00221-s001.docx]

Table S1. The primer sequences of seven *DlARFs* and reference gene in longan

| Primer name | **Sequence（ 5′ → 3′）** | **Primer name** | **Sequence（ 5′ → 3′）** |
| --- | --- | --- | --- |
| DlARF1-up | AGATTAGATGCCTCCATGCTTG | *DlARF9*-up | GCGACACTAGCACACATGGT |
| DlARF1-down | AAGGGAAGTATGACCAGGTTTG | *DlARF9*-down | CCATCCAGTAGTCAGAAGGTGC |
| DlARF2-up | GGAACTCGTAGCAAAGGATCT | *DlARF11*-up | GCATCTGATACAAGCACTCATGGT |
| DlARF2-down | TCTCAGTCCACCATCTTCACC | DlARF11-down | AACACACTCCACCCAGTAGTAAGC |
| DlARF6-up | GCTGAACCTGATACTGATGAG | DlARF16-up | GCCTCCAATAGAAGCTCATTTAC |
| DlARF6-down | CGAGGCAGTCAACGTCTTACAG | *DlARF16*-down | GCTCAATGGATCCAAGTCACTA |
| DlARF8-up | GACATTTCCAAGTTCAGCAGC | Actin-up | TTCCGCTGCCCAGAAGTCCTCTT |
| DlARF8-down | GTCATCACCAAGGAGAAGAACA | Actin-down | CATTGAACATAGTTGAACCACCACTGAG |

Table S2. Analysis of cis-elements in flower bud differentiation related ARF genes in longan

| Cis-elements name | | Site Function | DlARF1 | DlARF2 | DlARF6 | DlARF8 | DlARF9 | DlARF11 | DlARF16 |  |  |
| --- | --- | --- | --- | --- | --- | --- | --- | --- | --- | --- | --- |
| TGA-element | | auxin-responsive element | |  |  | 3 | 1 | 1 |  | 1 |  |
| AuxRR-core | | |  |  |  |  | 1 | 1 |  |  |  |
| CGTCA-motif | | | MeJA-responsiveness | |  |  |  | 1 | 4 | 1 | 1 |
| TGACG-motif | | |  |  |  |  | 1 | 1 | 4 | 1 | 1 |
| GARE-motif | | gibberellin-responsive | 1 |  | 1 |  |  |  |  |  |  |
| ABRE | | abscisic acid responsiveness | 2 | 1 | 2 |  | 2 |  | 2 |  |  |
| ARE | | anaerobic induction | 6 | 2 | 4 | 7 | 4 | 6 | 5 |  |  |
| circadian | | circadian control |  |  | 1 | 1 | 1 | 1 |  |  |  |
| TCA-element | | salicylic acid responsiveness | 2 |  | 2 |  |  |  | 1 |  |  |
| GCN4_motif | | in endosperm expression |  | 1 |  |  | 1 |  |  |  |  |
| CAT-box | | meristem expression |  |  |  |  |  | 1 |  |  |  |
| MSA-like | | cell cycle regulation |  |  |  |  |  |  | 1 |  |  |
| O2-site | | zein metabolism regulation | 1 | 1 |  |  |  |  | 2 |  |  |
| RY-element | | seed-specific regulation | 1 |  |  |  |  |  |  |  |  |
| MBS | | drought-inducibility | 2 |  |  |  |  | 1 |  |  |  |
| TC-rich repeats | | defense and stress responsiveness |  |  | 1 |  |  |  |  |  |  |
| LTR | | low-temperature responsiveness | 2 |  |  | 1 | 1 |  |  |  |  |

Figure S1. Protein sequences alignment between CiARF14 and DlARF13

The protein of CiARF14 (Cs7g02210.1) download from http://citrus.hzau.edu.cn/orange/index.php, sequences alignment used by DNAMAN8.0, the identity is 34.75%.
